# Expression dynamics of metalloproteinases during mandibular bone formation: association with Myb transcription factor

**DOI:** 10.3389/fcell.2023.1168866

**Published:** 2023-08-28

**Authors:** S. Varadinkova, V. Oralova, M. Clarke, J. Frampton, L. Knopfova, H. Lesot, P. Bartos, E. Matalova

**Affiliations:** ^1^ Laboratory of Odontogenesis and Osteogenesis, Institute of Animal Physiology and Genetics, v.v.i, Academy of Sciences, Brno, Czechia; ^2^ Department of Physiology, University of Veterinary Sciences, Brno, Czechia; ^3^ Institute of Cancer and Genomic Sciences, University of Birmingham, Birmingham, United Kingdom; ^4^ Department of Experimental Biology, Faculty of Science, Masaryk University, Brno, Czechia; ^5^ International Clinical Research Center, St. Anne’s University Hospital, Brno, Czechia

**Keywords:** metalloproteinases, MYB transcription factor, mandibular alveolar bone, development and remodelling, osteogenesis

## Abstract

As the dentition forms and becomes functional, the alveolar bone is remodelled. Metalloproteinases are known to contribute to this process, but new regulators are emerging and their contextualization is challenging. This applies to Myb, a transcription factor recently reported to be involved in bone development and regeneration. The regulatory effect of Myb on *Mmps* expression has mostly been investigated in tumorigenesis, where Myb impacted the expression of *Mmp1*, *Mmp2*, *Mmp7*, and *Mmp9*. The aim of this investigation was to evaluate the regulatory influence of the Myb on *Mmps* gene expression, impacting osteogenesis and mandibular bone formation. For that purpose, knock-out mouse model was used. Gene expression of bone-related *Mmps* and the key osteoblastic transcription factors Runx2 and Sp7 was analysed in Myb knock-out mice mandibles at the survival limit. Out of the metalloproteinases under study, Mmp13 was significantly downregulated. The impact of Myb on the expression of *Mmp13* was confirmed by the overexpression of Myb in calvarial-derived cells causing upregulation of *Mmp13.* Expression of *Mmp13* in the context of other Mmps during mandibular/alveolar bone development was followed *in vivo* along with *Myb, Sp7* and *Runx2*. The most significant changes were observed in the expression of *Mmp9 and Mmp13*. These MMPs and MYB were further localized *in situ* by immunohistochemistry and were identified in pre/osteoblastic cells as well as in pre/osteocytes. In conclusion, these results provide a comprehensive insight into the expression dynamics of bone related Mmps during mandibular/alveolar bone formation and point to Myb as another potential regulator of Mmp13.

## 1 Introduction

Matrix metalloproteinases (Mmps) are enzymes involved in many biological processes such as morphogenesis, angiogenesis, and wound healing, as well as related disorders including atheroma, arthritis, ulceration, and cancer (Visse and Nagase, 2003). Mmps have been recently recognized as biomarkers in several fields, including diagnosis or monitoring of treatment efficacy (Laronha and Caldeira, 2020). Mmps can be categorized into five types through bioinformatic analysis. Additionally, they can be subdivided based on substrate specificity, sequential similarity, domain organization structure, and biochemical properties, resulting in collagenases (incl. Mmps 1, 8, 13), gelatinases (incl. Mmps 2, 9), stromelysins (incl. Mmps 3, 10, 11), matrilysins (incl. Mmp 7), membrane-bound Mmps (incl. Mmp 14, 15, 16) and others (Page-McCaw et al., 2007; Young et al., 2019). Despite the relatively broad knowledge about Mmps, further research will be necessary to investigate the complexity of their regulation and activity. This applies also for osteogenesis, where Mmps play an important role in bone formation and remodelling (Fernandez-Patron et al., 2011). Notably, Mmp2, Mmp9, Mmp13, Mmp14 and Mmp16 have been reported as the most functionally important bone-associated Mmps in osteogenesis (Liang et al., 2016). Furthermore, Mmp2, Mmp9 and Mmp13 have also been emphasized in bone regeneration efforts (Khoswanto, 2023).

Several transcription factors known to regulate Mmps in bone have been identified, such as Sp7 and Runx2 (Zhang et al., 2012; Bruderer et al., 2014) but other potential candidates also exist. One of them is Myb, a protooncogene widely studied in cancerogenesis, haematopoiesis, and related disorders (Cicirò and Sala, 2021). Originally, Myb was associated with proliferating and undifferentiated cells but later it was identified also in differentiated cells (Ess et al., 1999; Soza-Ried et al., 2010). The latter case applies also for physiological chondrogenesis and osteogenesis. Myb was reported as a regulator of osteoclastogenesis via the system Rankl/Opg (Takanche et al., 2018; Kodric et al., 2019) and of the extracellular matrix (ECM) production (Matalova et al., 2011; Oralova et al., 2015; 2017).

Given that Mmps play a pivotal role in the remodelling of the bone extracellular matrix, and considering Myb regulates ECM factors, it is plausible that Mmps may be involved in the osteogenic effects by Myb. The background for such an expectation arises from observations made in cancer cells (Bhattarai et al., 2011; Knopfova et al., 2012; Xu et al., 2019), but have not yet been investigated in osteogenesis.

Osteogenesis includes a tight interplay among the key bone cell types: osteoblasts, osteocytes, and osteoclasts. Consequently, investigating osteogenesis *in vivo* is highly favoured. Therefore, we took advantage of the Myb knock-out mouse model to test the hypothesis that Myb could impact the expression of Mmps during bone formation and remodelling. Unfortunatelly, the survival of Myb knock-out is limited by the prenatal day 15 (Mucenski et al., 1991). At this point, long bones are only at the chondrogenic stage while the intramembranous mandibular/alveolar bone already contains all three bone cell types (reviewed in Svandova et al., 2020).

The analysis of *Mmps mRNA* expression (*Mmps 1, 2, 3, 7, 8, 9, 10, 11, 12, 13, 14, 15* and *16*) was performed on Myb knock-out mandibular samples and was followed by overexpression experiments in a calvaria-derived osteoblastic cells *in vitro*. Additionally, complex dynamics of Mmps expression at the onset of mandibular/alveolar bone formation (around E15) was provided to fill in missing gaps in mandibular/alveolar bone development characterization. The localization of MYB protein and selected MMPs (MMP9 and MMP13) in the forming mandibular/alveolar bone was performed by immunohistochemistry (IHC) to assess their temporo-spatial pattern.

## 2 Materials and methods

### 2.1 Animals

Wild type mice (strain CD1) were purchased from the Breeding Units of Masaryk University, Brno, Czech Republic. Heads corresponding to the embryonic days 13, 14, 15, 18 (E13, E14, E15, E18) and postnatal day (P2) were collected. The work with laboratory animals in the CR, including euthanasia, is regulated by Act No. 359/2012. According to the paragraph 3t), *post mortem* collection of organs and tissues is not considered as an experiment.

Myb-deficient mice (Mucenski et al., 1991 on a CB7BL/6 background) were maintained at the Institute of Cancer and Genomic Sciences, University of Birmingham, United Kingdom. Heads of wild type (WT) or knock-out (KO) embryos were collected prior to their survival limit at E15. The work with laboratory animals in Birmingham was performed under the terms of a UK Home Office licence according to the Animals (Scientific Procedures) Act, 1986.

### 2.2 Separation of the molar region

Mouse mandibles at E13, 14, 15, and 18 (CD1 strain) were dissected from freshly collected samples. The molar region together with the alveolar bone were removed from mandibles using micromanipulation (as described in Vesela et al., 2019). At least 3 embryos were used for each stage, and both molar regions (right and left sides) were dissected from each mandible. The tissues were lysed in the RLT buffer (Qiagen) with 1% 2-mercaptoethanol (Sigma-Aldrich, M3148-100 ML) and used for the subsequent isolation of RNA.

Mandibular molar regions of the wild type (WT) and Myb knock-out (KO) mouse embryos (survival limit E15) were dissected. At least 3 embryos were used for WT and KO with both molar regions (right and left sides). Dissected samples were prepared for RNA extraction and subsequent qRT-PCR analysis.

### 2.3 Cell line

Murine calvaria cells were obtained from the calvaria of neonatal CD1 mice 2 days after birth by sequential collagenase digestion at 37°C with minor changes in protocol described in Garcia et al. (2002). Samples from 12 mice were used for calvarial cell isolation to obtain a sufficient amount of cells. Briefly, for transfection experiments, the calvaria-derived cells were cultured until 80% confluence and the proliferation medium was replaced by a differentiation medium (α-MEM containing 10% FBS, 2 mM glutamine, 50 μg/mL ascorbic acid, and 10 mM β-glycerolphosphate). The culture medium was replaced every 3 days. The biological samples were collected at 24 h and 14 days for investigation.

### 2.4 Over expression of Myb in calvaria cells

The murine calvaria cells were transfected with the Myb-expressing vector (pcDNA3-mcMYB) or control vector (pcDNA-3), as was described earlier in Oralova et al., 2015; Oralova et al., 2017. The plasmids were added at day 0 and 7, respectively in the concentration of 100 ng DNA per 10 µL of cells using the FuGENE HD 6 transfection reagent (Cat. No. E2311, Promega, United States). The medium was changed every 2–4 days, cultures were collected at 24 h and 14 days.

### 2.5 qRT-PCR

Total RNA was extracted from the molar part region, calvaria and IDG-SW3 cells using the RNeasy Mini Kit (Qiagen, Germany). RNA concentration and purity were assessed using a NanoDrop and cDNA was synthesized using the reverse Master Mix (Generi Biotech, Czech Republic). For cDNA synthesis, 500 ng of RNA was used, and biological replicates of each sample were diluted to a concentration of 100 ng/μL before performing a qRT-PCR reaction. The qRT-PCR was performed in a final reaction volume of 10 μL containing the one-step Ideal PCR Master Mix (Generi Biotech, Czech Republic) using Light-Cycler 96 (Roche, Switzerland) with preheating to 95 °C for 10 min. This was followed by 40 cycles of 95 C/15 s and 62.5°C/1 min with mRNA probes for *Mmp1a, Mmp1b, Mmp2, Mmp3, Mmp7, Mmp8, Mmp9, Mmp10, Mmp11, Mmp12, Mmp13, Mmp14, Mmp15, Mmp16, Myb, Runx2* and *Sp7* (Mouse *Mmp1a,* Mm00473485_m1; *Mmp1b*, Mm00473493_g1; *Mmp2*, Mm00439498_m1; *Mmp3*, Mm00440295_m1; *Mmp7*, Mm00487724_m1; *Mmp8*, Mm00439509_m1; *Mmp9*, Mm00442991; *Mmp10*, Mm01168399_m1; *Mmp11*, Mm00485048_m1; *Mmp12*, Mm00500554_m1; *Mmp13*, Mm00439491_m1; *Mmp14*, Mm00485054_m1; *Mmp15*, Mm00485062_m1; *Mmp16,* Mm01210646_m1; *Myb*, Mm00501741_m1; *Runx2* Mm00501584_m1*; Sp7* Mm00504574_m1 TaqMan Gene expression Assay, Thermo Fisher Scientific, United States). Expression levels were calculated using the ΔΔCT method, with normalization against β−*actin* RNA levels (mouse *Actb*, Mm02619580_g1, TaqMan Gene Expression Assay, Thermo Fisher Scientific, United States).

### 2.6 Histology

Mouse heads at E13, 14, 15, and 18 (CD1 strain) were dissected, fixed in 4% buffered paraformaldehyde, dehydrated in ethanol series, treated with xylene, and embedded in paraffin. Histological sections were processed for haematoxylin-eosin (H&E) staining and immunohistochemistry (IHC). Wild type (WT) and Myb knock-out (KO) mouse embryos were processed in the same way and prepared for histological analyses (haematoxylin-eosin; haematoxylin-eosin-alcian blue; Masson’s green trichrome; Weigert van Gieson; Von Kossa and Alizarin red).

### 2.7 Immunohistochemistry

Serial frontal histological sections (5 μm) were prepared for IHC staining. Citrate solution was used as a pre-treatment (5 min/98°C). Endogenous peroxidases were inhibited using 3% hydrogen peroxide for 5 min/RT. Blocking serum (Vectastain Elite ABC Kit, Rabbit Ig, PK-6101, Vector Laboratories, United States) was applied for 20 min/RT, primary antibodies against MYB (anti-MYB, 1:300, ab226470, Abcam), MMP9 (anti-MMP9, 1:100, PA5-13199, Thermo Scientific) and MMP13 (anti-MMP13, 1:100, ab39012, Abcam) were diluted in DAKO antibody diluent (Dako, S3022, North America) and applied for 1 h/RT. The secondary antibodies and avidin-biotin complex (Vectastain Elite ABC Kit, Rabbit Ig, PK-6101, Vector Laboratories, United States) were applied each for 30 min/RT. Tissue antigens were visualised by DAB (diaminobenzidine) chromogen substrate (Liquid DAB + Substrate, K3468, Dako North America, United States). Counterstaining was achieved using haematoxylin.

### 2.8 Myb-binding consensus sequences

The sequence of the Mmp13 promoter region was scanned for the presence of potential Myb-binding sites using a consensus motif of YAAC^T/^(C)_/G_GYCR derived by selection of oligodeoxynucleotides bound to mouse Myb protein *in vitro* (Howe and Watson, 1991).

### 2.9 Statistical analysis

All results were expressed as mean ± standard deviations (SD) of four samples for each time point and compared using an unpaired *t*-test (unpaired *t*-test, two-tailed, significance level = 0.05) and one-way ANOVA (One-way analysis of variance, Tukey test: Compare all pairs of columns, Significant level = 0.05). Differences were considered as significant at *p* < 0.05; *p* < 0.01; *p* < 0.001 and are indicated by *; **; *** symbols, respectively.

## 3 Results

### 3.1 Expression of bone-associated *Mmps*, *Sp7,* and *Runx2* in the Myb-deficient mandible

First, the expression profile of bone-related Mmps was compared in Myb-deficient and wild type mouse mandibles (prior to the survival limit around the E15). Among investigated Mmps, Mmp13 was significantly downregulated.

**FIGURE 1:**
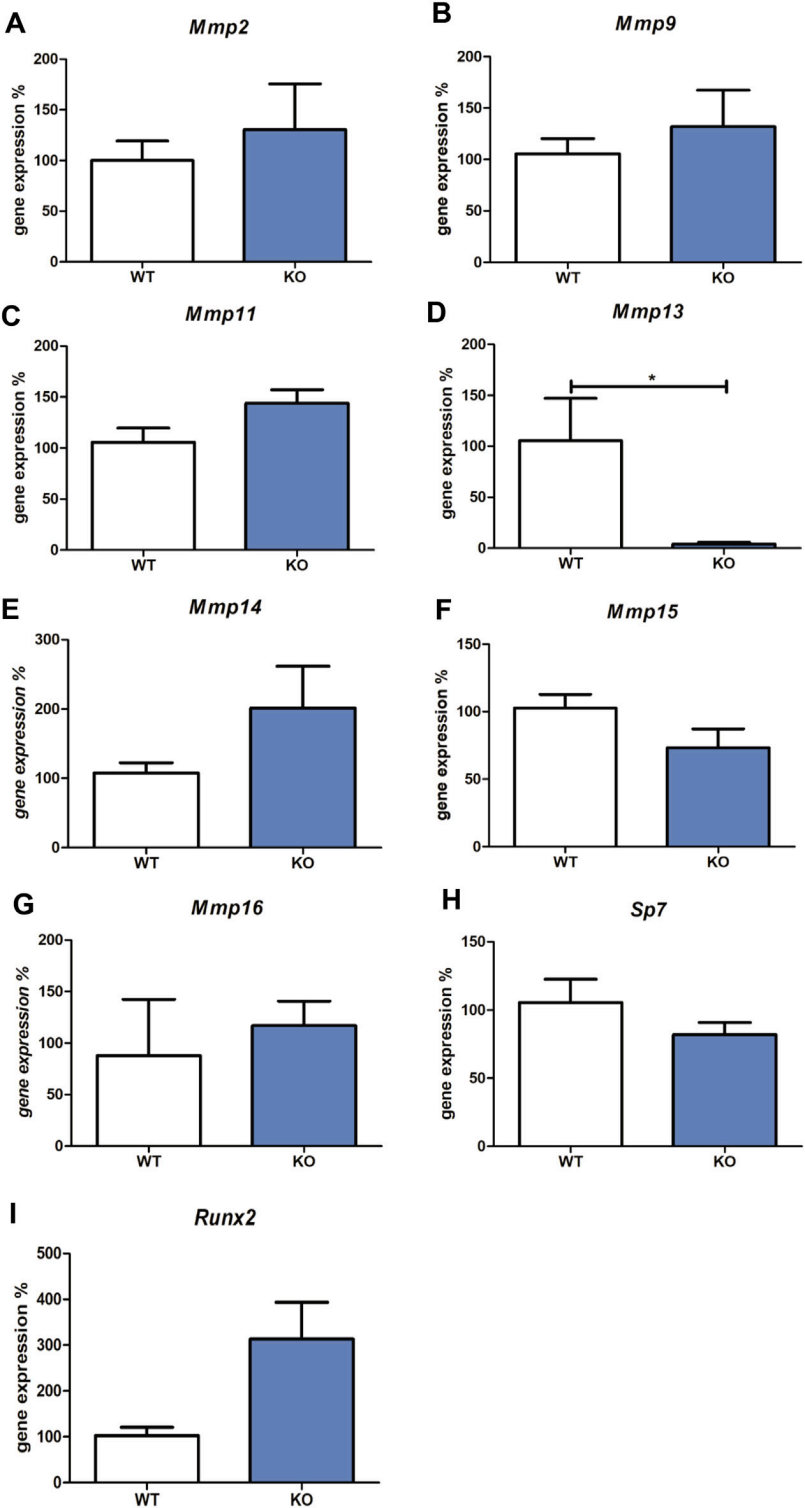
Expression of *Mmp2, Mmp9, Mmp11, Mmp13*, *Mmp14, Mmp15, Mmp16, Sp7 and Runx2 m*RNAs. The graph shows expression of mRNAs corresponding to *Mmp2* **(Panel A)** *, Mmp9* **(Panel B)** *, Mmp11* **(Panel C)** *, Mmp13* **(Panel D)** *, Mmp14* **(Panel E)**, *Mmp15* **(Panel F)**, *Mmp16* **(Panel G)**, *Sp7* **(Panel H)**, and *Runx2* **(Panel I)** in tissues derived from wild type (WT) compared to Myb deficient mice (KO), detected by qRT-PCR. Data are expressed as mean ± SD (n—4 per group and statistically significant differences are highlighted (unpaired *t*-test, two-tailed, *p < 0.05).

The expression of all *Mmps* (*Mmp1a*, *Mmp1b*, *Mmp2, Mmp3, Mmp7, Mmp8, Mmp9, Mmp10, Mmp11, Mmp12, Mmp13, Mmp14, Mmp15,* and *Mmp16*) were detected in wild-type *versus* Myb knock-out embryos using qPCR. Transcripts of *Mmp1a*, *Mmp1b*, *Mmp3, Mmp7, Mmp8, Mmp10,* and *Mmp12* were not detected. The expression of *Mmp2, Mmp9, Mmp11, Mmp14, Mmp15,* and *Mmp16* was not significantly altered in *Myb* knock-out tissues compared to the wild-type (shown in Figures 1A–C,E–I). *Mmp13* expression was significantly decreased in *Myb* mutant embryos (0.1-fold change compared to wild type, shown in Figure 1D).

Since the most well-known regulators of Mmp13 expression are Sp7 and Runx2, expression levels of these two factors were analysed in Myb-deficient *versus* wild type samples. Osteoblastic *Sp7* and *Runx2* transcription factors mRNA levels were not significantly altered in *Myb* knock-out tissues compared to the wild-type (shown in Figures 1H,I).

Histological analysis of the mandibular/alveolar bone in Myb-deficient mice did not show major differences (Supplementary Figure S1), however Myb-deficient mice tend to be smaller than their littermates.

### 3.2 Myb over expression in osteoblastic cells upregulates *Mmp13* transcription

At the survival limit of the Myb-deficient mice, osteoblasts are the major population of cells within the mandible. Therefore, osteoblastic cells of intramembranous origin (isolated from calva, described in Material and Methods) were used to confirm the opposite effect of Myb on Mmp13 expression. Over expression was performed in two experimental settings (Supplementary Figure S1A,B). The immediate effect of Myb over expression was evaluated after 24 h, the long-term effect after 14 days.

**FIGURE 2:**
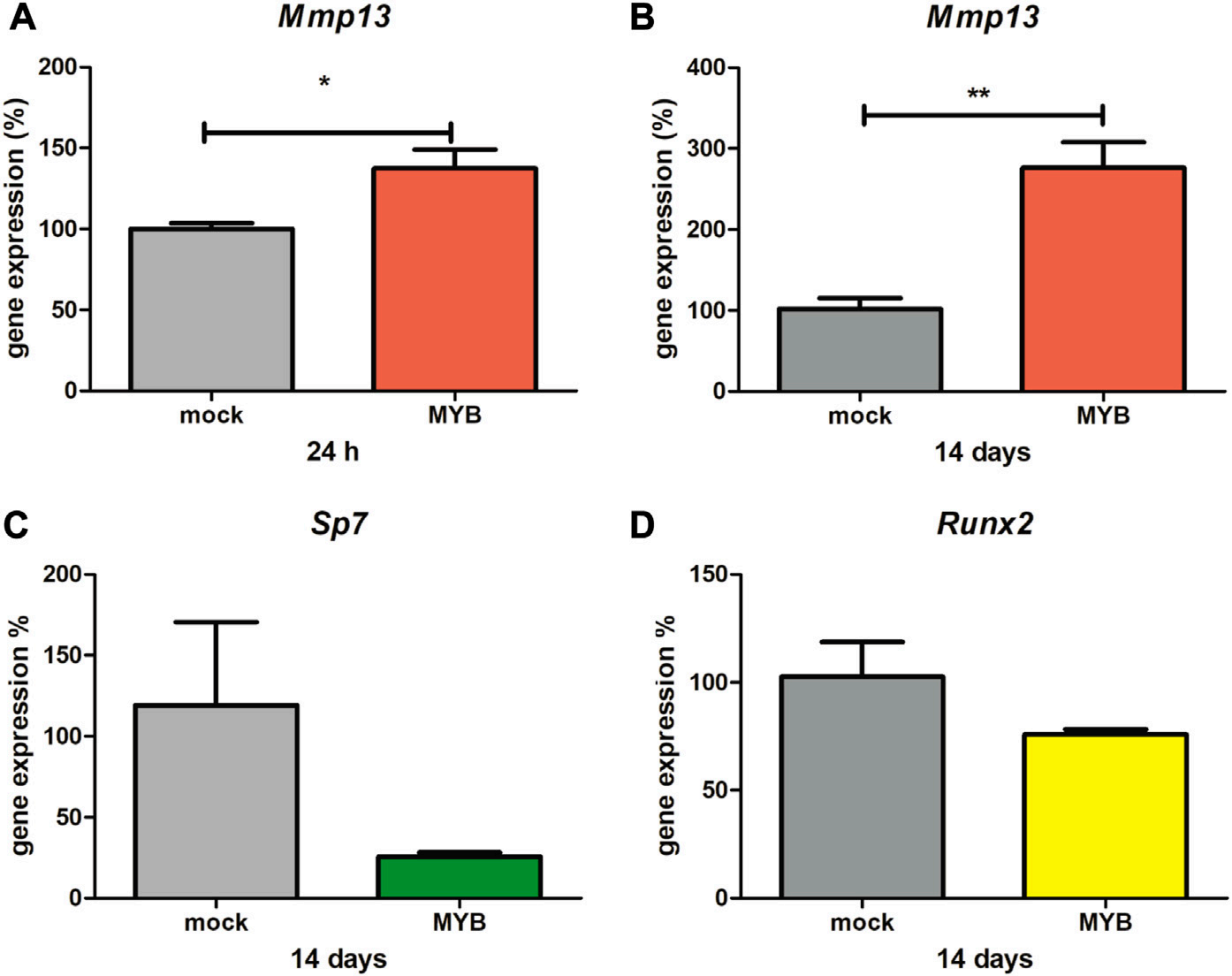
*Mmp13, Sp7* and *Runx2* expression in overexpressed calvaria cell line. The graph shows calvaria cells, treated by Myb vector (MYB) and empty vector (mock), expression of *Mmp13* at 24 h **(Panel A)** and 14 days **(Panel B)**, expression of *Sp7* at 14 days **(Panel C)** and expression of *Runx2* at 14 days **(Panel D)**. Data are expressed as mean ± SD (n—4 per group) and statistically significant differences are highlighted (unpaired *t*-test, two-tailed, *p < 0.05; **p < 0.01).

The *Mmp13* expression significantly increased in both cases, 24 h (1.4-fold change compared to control, shown in Figure 2A) and 14 days (2.8-fold change compared to control, shown in Figure 2B). The expression of osteoblastic *Sp7* (Figure 2C) and *Runx2* (Figure 2D) transcription factors was not significantly altered in the Myb over expressing calvaria cell line.

Therefore, we looked for the presence of potential Myb-binding sequence motifs (MBS) in the Mmp13 promoter, using the consensus sequence YAAC^T/^(C)_/G_GYCR (Supplementary Figure S2). Taking the YAAC element of the consensus motif as being essential, 2 sequences with a 7 out of 9 match can be seen, with a further three motifs with a 6 out of nine alignments.

### 3.3 The expression profile of *Mmps, Myb, Sp7, and Runx2* in the molar part region during mouse prenatal development

In order to investigate the expression profile of bone-related Mmps, Sp7 and Runx2 in mandibular development *in vivo*, stages around the survival limit of the Myb knock-out mice were examined. The prenatal stages E13-E15 correspond to the onset of mandibular bone formation when osteoblasts, osteoclasts, and osteocytes keep appearing. By day 18, all three major cell populations become established.

qRT-PCR was used to provide the expression data for *Mmp2, Mmp9, Mmp11, Mmp13, Mmp14, Mmp15,* and *Mmp16*. As in the case of the Myb knock-out and corresponding wild type samples, expression of *Mmp1a, Mmp1b, Mmp3, Mmp7, Mmp8, Mmp10,* and *Mmp12,* was not detected in the molar area at any of the time points investigated.

**FIGURE 3:**
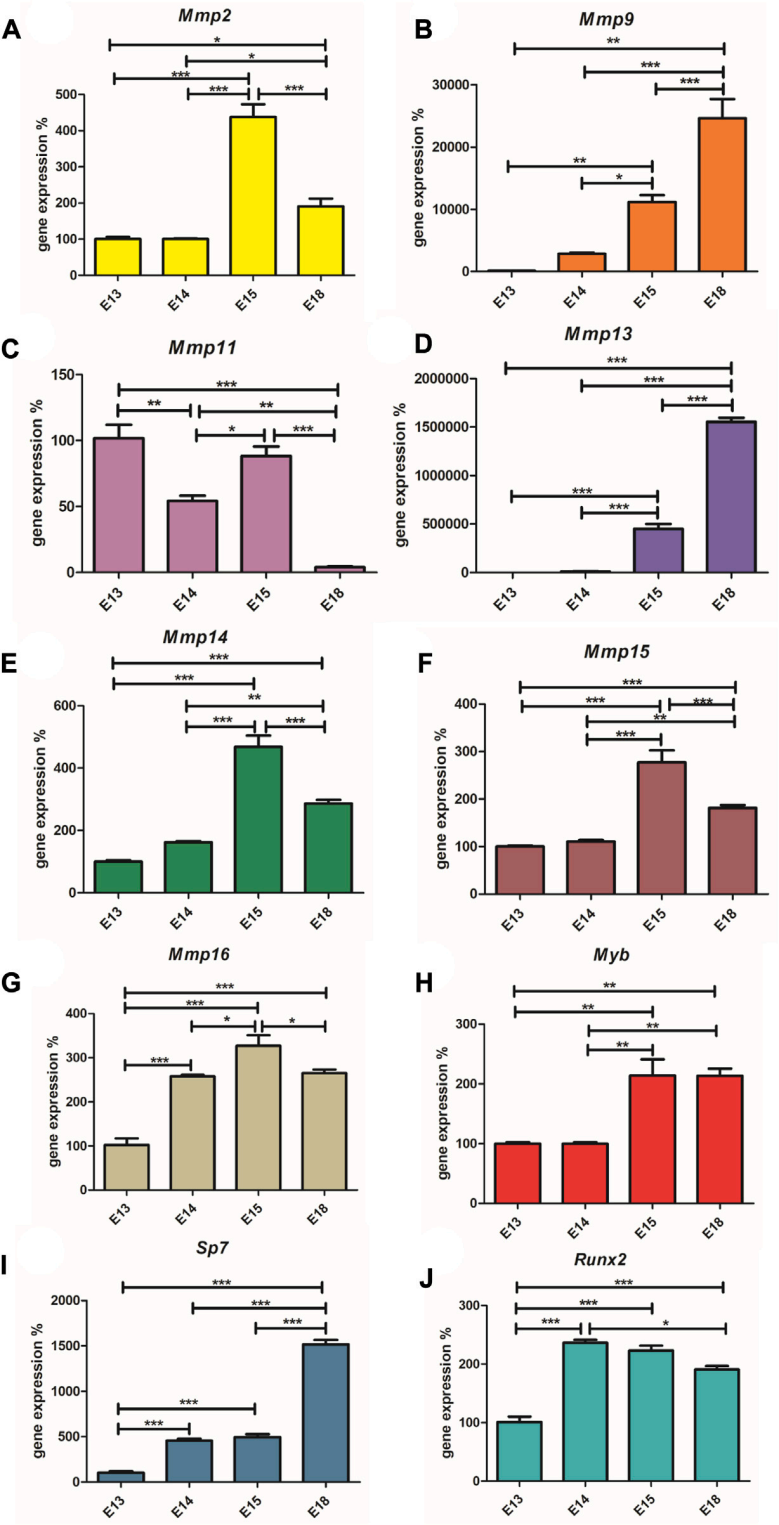
Expression (mRNA) of *Runx2, Sp7, Myb, Mmp2, Mmp14, Mmp15, Mmp16, Mmp11, Mmp9, and Mmp13*. The graph shows expression of mRNAs corresponding to *Runx2* **(Panel A)** *, Sp7* **(Panel B)** *, Myb* **(Panel C)** *, Mmp2* **(Panel D)** *, Mmp14* **(Panel E)**, *Mmp15* **(Panel F)**, *Mmp16* **(Panel G)**, *Mmp11* **(Panel H)**, *Mmp9* **(Panel I)** and *Mmp13* **(Panel J)** during embryonic alveolar bone development detected by qPCR. Data are expressed as mean ± SD (n—4 per group) and statistically significant differences are highlighted (One-way ANOVA, Tukey test, *p < 0.05; **p < 0.01; ***p < 0.001).

*Runx2,* as a positive regulator of osteoblasts, is highly expressed at E14 (Figure 3A), when the first osteoblasts appear and then its expression decreases towards E18. Conversely, the expression of *Sp7*, the transcription factor of mature osteoblasts, increases along with the number of these cells (Figure 3B). *Myb* expression was at the same level at E13 and E14, rapidly increased at E15 and remains at a high level at E18 (Figure 3C). *Mmp2, Mmp14, Mmp15, Mmp16* show a very similar pattern with increasing expression towards E15 and with a decreased level at E18 (Figures 3D–G). *Mmp11* has a variable expression during mandibular/alveolar bone development, nevertheless, with significantly decreasing expression until E18 (Figure 3H). Expression of *Mmp9* and *Mmp13* dramatically increases throughout bone development at E18 (Figures 3I,J). The statistical significance (*p* < 0,05; *p* < 0,01; *p* < 0.001) of each expression change is indicated in the figures by symbols *, **, ***, respectively.

### 3.4 Immunohistochemical localization of MYB and MMPs during mandibular/alveolar bone formation

To detect the protein expression of the investigated MYB and MMPs factors *in situ*, immunohistochemistry was applied.

**FIGURE 4:**
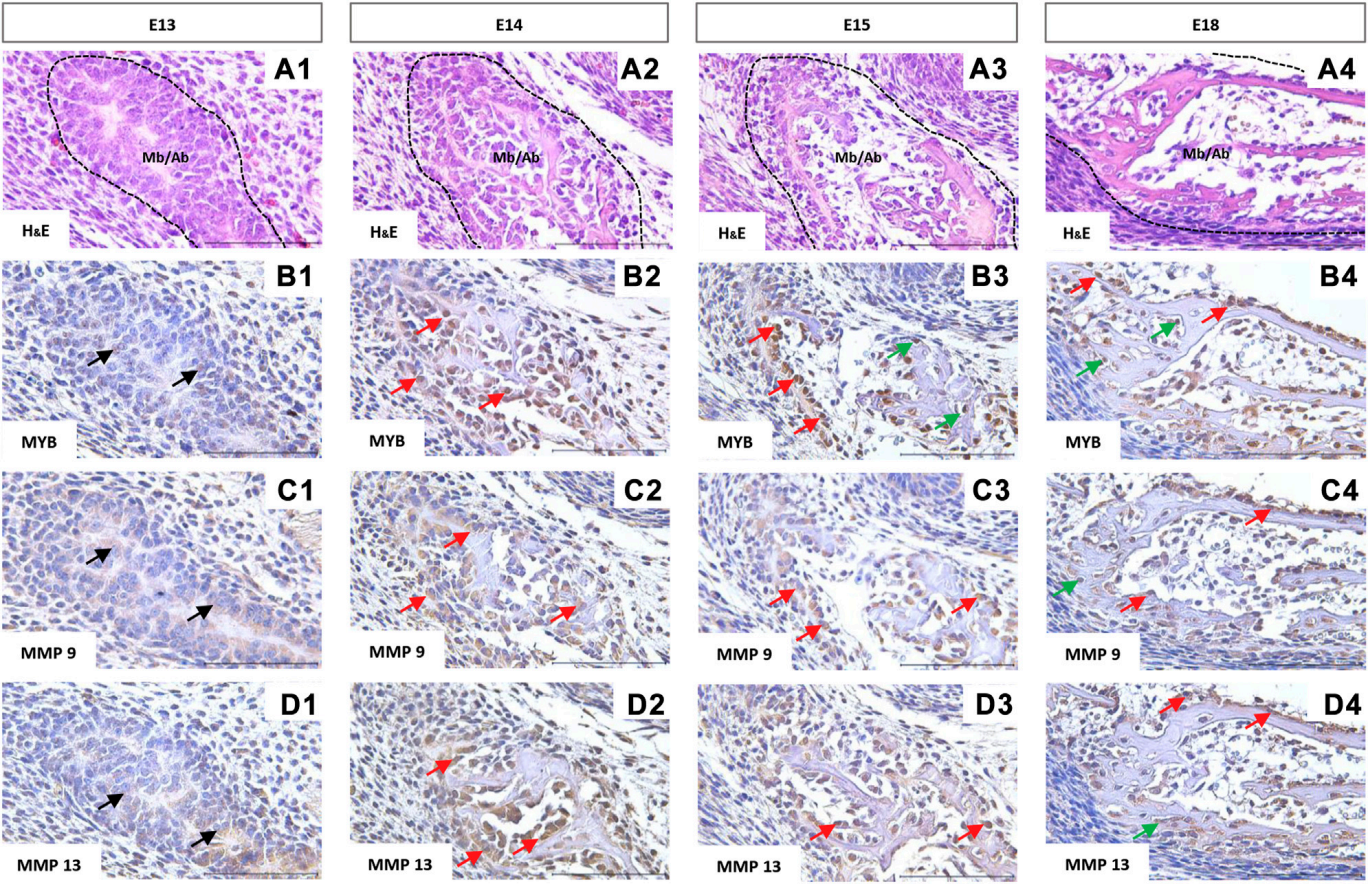
Immunohistochemical localization of MYB **(B1–B4)**, MMP9 **(C1–C4)**, and MMP13 **(D1–D4)** proteins in the forming mandibular/alveolar bone. Embryonic day (E) 13, 14, 15, 18. Haematoxylin and eosin (H&E) staining **(A1–A4)**. Arrows point to the examples of positive cells: black = mesenchymal cells, red = osteoblasts, green = osteocytes. Scale bar =100 μm.

The first structure of the mandibular/alveolar bone is morphologically visible at prenatal day 13, where the mesenchymal cells condense and differentiate into osteoblasts (Figure 4A1). MYB-positive nuclei were observed at this stage (Figure 4B1). At E14, MYB was localized in osteoblasts (Figure 4B2), and both osteoblasts and osteocytes were MYB-positive at E15 and E18 (Figure 4B3,B4).

Matrix metalloproteinases 9 and 13 showed similar expression in histological sections. Both MMPs were expressed by the mesenchymal cells (Figure 4C1, 4D1) and were localized especially in the cell cytoplasm (Figures 4C,D), however positive nuclei were also observed in the case of MMP13 (Figure 4D2,D3,D4). At the stage E18, MMP9 and 13 positive osteocytes were found (Figure 4C4,D4).

## 4 Discussion

The comprehensive exploration of matrix metalloproteinases and mechanisms governing their multilevel regulation is challenging to understand tissue homeostasis (Gaffney et al., 2015). But, also to try to interconnect molecular biology, physiology, pathophysiology, and pharmacology in order to develop new selective therapeutic agents against various diseases (Vise and Nagase, 2003).

The Myb is essential for hematopoiesis (Soza-Ried et al., 2010) and its classical roles apply for undifferentiated and cancer cells (Cicirò and Sala, 2021). More recently, Myb has been linked to differentiated cells (Sandberg et al., 2005) including bone related ones (Matalova et al., 2011; Oralova et al., 2015; 2017). The challenge of analyzing Myb knock-out mice in osteogenesis is hindered by the prenatal lethality, by the day 15 in the mouse (Mucenski et al., 1991). This study capitalizes on Myb-deficient mice mandibles as a unique model to unravel the osteogenic potential of Myb transcription factor.

*In vitro* models are favoured in compliance with 3R principles to reduce or replace animal testing (Robinson et al., 2019). However, despite a rapid progress in the field (Owen and Reilly, 2018; de Wildt et al., 2019) there are no established *in vitro* models for bone remodelling (Ehnert et al., 2020). Therefore, to cover the cellular and extracellular complexity of the bone, animal models are extremely important particularly for translational research (Berridge, 2021). The mouse model is used for it is showing many similarities to humans, relatively short reproductive period, and financial availability. In addition, a wide spectrum of information is available at the genome, transcriptome, and proteome levels (Elefteriou and Yang, 2011).

This investigation provides data directly from the *in vivo* context of mandibular/alveolar bone formation. Metalloproteinases are essential for bone remodelling as well as homeostasis (Cicirò and Sala, 2021). Notably, the identification of some Mmps (Mmp1, Mmp2, Mmp7 and Mmp9) has been reported as Myb-regulated genes in cancer cells (Knopfova et al., 2012; Xu et al., 2019). At the stage of Myb knock-out lethality (by prenatal day 15), *Mmps 2, 9, 11, 13, 14, 15* and *16* transcripts were detected in the mandibular samples. Out of these, expression of *Mmp13* was significantly downregulated in the Myb knock-out mandibles. Mmp13, primarily secreted by chondrocytic and osteoblastic cells (Sasano et al., 2002), is the crucial collagenase that cleaves ECM collagens during bone and cartilage development (Stickens et al., 2004) especially collagen type I (Takaishi et al., 2008). As Mmp13 is important for bone remodelling (Tang et al., 2012), we can therefore speculate that the decreased Mmp13 expression in the Myb knock-out mice might reverberate within the bone tissue’s collagen type I breakdown. Interestingly, potential several Myb binding sites within the Col1a2 promoter suggests that it might be involved in the regulation of collagen type I production (reviewed in Jinnin, 2010). Mmp13 is well-known to be required for proper fracture healing (Nishimura et al., 2012; Tang et al., 2012). This may also apply for the intramembranous bone and could even be associated with the effect of Myb during dental implant osseointegration (Bhattarai et al., 2013). Mmp13 is an important marker of mature osteoblasts as demonstrated in the long bones (Zhang et al., 2012). Mmp13-deficient mice demonstrated abnormal bone phenotypes and increased trabecular bone volume (Stickens et al., 2004).

The precise regulation of Mmp13 expression in osteoblasts has not yet been completely elucidated. However previous studies have indicated that the transcription factors Runx2 and Sp7 (osterix) contribute to Mmp13 transcription (Zhang et al., 2012; Komori, 2018; Liu et al., 2020). Our investigation revealed that neither Runx2 nor Sp7 exhibited any significant alterations in the Myb-deficient mandible. This observation suggests the potential involvement of Myb as another regulator in bone development. This hypothesis gains support from the identification of Myb binding sites (MBS) within the Mmp13 promoter. Furthermore, overexpression of Myb in calvaria-derived cells confirmed that there were no significant changes in expression of Runx2 or Sp7, despite Mmp13 was upregulated. Notably, dysregulated overexpression or activation of Mmp13 has been associated with the excessive bone tissue breakdown and contribution to the pathogenesis, e.g., in the case of osteoarthritis (Hu and Ecker, 2021). Furthermore, the specific pattern of expression changes in Mmp13 could provide insights into the regulatory mechanism by which Myb influences Mmp13 expression. Thus, the increased in Mmp13 expression induced by Myb may occur through direct binding to the Mmp13 promoter region.

To contextualize the findings with mandibular/alveolar bone formation process, and the critical period of Myb knock-out lethality, different developmental stages of the mandible were investigated. From prenatal days 13–15 (E13-E15), the first osteoblasts appear (E13), followed by influx of osteoclasts (E14) and differentiated osteocytes (E15) (Alfaqeeh et al., 2013).

Along with *Mmp 2*, *9, 13, 14* and *16* identified in long bone development (Liang et al., 2016), *Mmp11* and *15* mRNAs were detected in the mandibular samples. The other Mmps (Mmp 1,3,7,8,10,12) displayed negligible expression.

The expression profiles of *Mmp 2, 14, 15* and *16* peaked around day 15. Mmp2 is involved in the formation and maintenance of the osteocyte channel network (Inoue et al., 2006) initiated around this stage. Remarkably, the expression pattern and levels of this metalloproteinase were identical with that of Mmp14. Mmp14 was reported as a novel target of PTH (and RANK/RANKL/OPG) signalling, which applies for osteoblasts as well as osteocytes (Delgado-Calle et al., 2018). Therefore, the elevation is likely to be connected with initiated osteocytogenesis and bone remodelling at the onset of mandibular/alveolar bone establishment. Not much is known about the function of Mmp15 in bone (Fatima et al., 2020). Mmp16 is present in osteoblasts and osteocytes (Haeusler et al., 2005) and is important for their viability (Shi et al., 2008). Thus, the elevation in Mmp16 could again correspond with the differentiation of first osteocytes at this stage.

Mmp11 was the only one among the metalloproteinases investigated that exhibited decreasing expression levels during the onset of alveolar bone formation. In general, Mmp11 plays a role in whole body metabolism and energy homeostasis (Cabral-Pacheco et al., 2020). Mmp11 has been associated with many physiological processes where ECM remodelling takes place, such as embryonic development (Klein and Bischoff, 2010). The physiological role of Mmp11 is unclear, but unlike the other Mmps investigated here, Mmp11 only weakly degrades structural proteins of the extracellular matrix (Reddy and Rani, 2017).

*Mmp13* (relative gene expression compared to *actin*) unveiled the most striking expression changes within the developmental dynamics and also its relative levels (compared to other Mmps). Beyond its function in bone remodelling, Mmp13 plays multiple roles in osteogenesis (Nakatani and Partridge, 2017), including the formation of osteocytic processes and networks (Tang et al., 2012), and association with PTH pathways has been noted (Lee and Partridge, 2010). A pivotal study by Behonick et al. (2007) further underscores the significance of Mmp13 in bone physiology. Their research on Mmp13^−/−^ mice revealed disrupted bone remodelling during the healing of stabilized fractures and cortical defects via intramembranous ossification. Specifically, the study found that Mmp13^−/−^ mice had delayed bone healing and reduced bone formation compared to the control mice (Behonick et al., 2007). This demonstrated that Mmp13 is involved in the normal remodelling of bone and cartilage during adult skeletal repair, suggesting a direct involvement of Mmp13 in the initial stages of ECM degradation. Therefore, based on the broad impact of Mmp13 on both early but also on later osteogenesis, it is plausible to speculate that the consequence of Myb deficiency could extend to various stages of bone development. However, due to the prenatal lethality, this cannot be followed using the Myb knock-out mice analysed in the presented research.

Another Mmp with remarkable changes in expression dynamics during development was *Mmp9*. Mmp9 is critical for long bone development since it prevents accumulation of late hypertrophic chondrocytes in the growth plate (Liang et al., 2016). In early bone development, Mmp9 is expressed by osteoclasts (Reponen et al., 1994) appearing in the mandibular/alveolar bone at E14, when the expression of *Mmp9* keeps a gradual increase towards E18. This increase also corresponds to the onset of angiogenesis within the bone region. Mmp9 has been demonstrated to participate in angiogenesis (Cackowski et al., 2010) and was investigated extensively also in cancerogenesis (Vu and Werb, 2000).

This study aimed to investigated the localization of Myb protein in osteoblasts, osteocytes and osteoclasts during the prenatal period of mandibular/alveolar bone formation/remodelling was provided. The first reports about MYB protein in alveolar bone development were provided by our team earlier (Matalova et al., 2011; Lungova et al., 2012). Notably, MMP9 and MMP13 proteins colocalized within the Myb-positive osteoblasts, osteocytes and osteoclasts. These two Mmps observed a coincidence with the most significantly increasing mRNA expression levels, as determined by RT-qPCR. This observed co-localization of MYB, MMP9, and MMP13 proteins provides additional support to the hypothesis about the Myb transcription factor being involved in the regulation of Mmps expression dynamics (Paiva and Granjeiro, 2017).

In the initial steps of alveolar bone development, metalloproteinases play intricate roles in regulating osteoblast/osteocyte differentiation, orchestrating bone formation and resorption, and facilitating osteoclast recruitment and migration via bloodstream (Omi and Mishina, 2022). This dynamic interplay is fundamental for establishing the alveolar bone as an integral part of the periodontal apparatus anchoring the tooth within the jaw and as such being subjected to regeneration therapies (Ward, 2022). Our findings are in agreement with the Myb contribution to osseointegration of dental implants as reported before (Bhattarai et al., 2013). The significant downregulation of Mmp13 expression in Myb-deficient mice suggests the possibility of direct Myb transcription factor binding within the Mmp13 promotor, intricately intertwined with processes of bone formation and resorption.

The recent investigations thus underscored the role of Mmps in the context of periodontium. The identification of novel components within the regulatory networks governing Mmps expression and functionality holds promise for enhancing our understanding of these processes and advancing strategies for the prevention and treatment of periodontal diseases (Checchi et al., 2020; Luchian et al., 2022).

It is worth mentioning that a number of pathologies are associated with impaired balance in bone remodelling due to altered expression/activation of Mmps, such as osteolysis, osteoarthritis, or osteoporosis (Paiva and Granjeiro, 2017). In cancerogenesis, upregulation of Mmp13 is associated with poor prognosis in oral squamous cell carcinoma (Vincent-Chong et al., 2014). Evidence about Mmp-13 in breast cancer-induced osteolysis indicates this Mmp as a promising therapeutic target for breast cancer bone metastasis (Kwon, 2023), where also Myb is involved as well (Knopfova et al., 2012).

In conclusion, our study has shed light on the impact of Myb on metalloproteinases, extending well beyond the immediate scope of our study. In this context, our study contributes to the broader scientific dialogue surrounding the intricate interplay between Myb and metalloproteinases in patho/physiological osteogenesis, opening research for further exploration and potential clinical applications. Therapeutic strategies aimed at regulating Mmp13 activity (Hu and Ecker, 2021), via the transcription factor Myb may be used for emerging treatments (Kou et al., 2021).

## Data Availability

The original contributions presented in the study are included in the article/Supplementary Materials, further inquiries can be directed to the corresponding author.

## Supplementary material

SUPPLEMENTARY FIGURE S1 Wild type (WT) and Myb knock-out (KO) mandibular/alveolar bone. The haematoxylin-eosin staining (H&E); haematoxylin-eosin-alcian blue staining (H&E/Alc); Masson’s green trichrome (MGT); Weigert van Gieson (WvG); Von Kossa (VKs) and Alizarin red (AlzR). Scale bar = 100 μm.

SUPPLEMENTARY FIGURE S2 Expression of Myb in overexpressed calvaria cell line. The graph shows expression of RNAs corresponding to Myb in calvarial cells treated by Myb vector (MYB) and empty vector (mock) at 24 h (Supplementary Figure S1A) and 14 days (Supplementary Figure S1B). Data are expressed as mean ± SD (n—4 per group) and statistically significant differences are highlighted (unpaired t-test, two-tailed, ***p < 0.001).

SUPPLEMENTARY FIGURE S3 Nucleotide sequence of Mmp13 promotor. Representation of the Mmp13 sequences upstream of the first exon (yellow), which contains the translation initiation codon (red). Sequences with a match to the YAACT/(C)/GGYCR consensus motif are highlighted, where cyan and pink represent 7/9 and 6/9 nucleotide matches, respectively.

## References

[B1] Alfaqeeh S. A. Gaete M. Tucker A. S. (2013). Interactions of the tooth and bone during development. J. Dent. Res. 92 (12), 1129–1135. 10.1177/0022034513510321 24155263

[B2] Behonick D. J. Xing Z. Lieu S. Buckley J. M. Lotz J. C. Marcucio R. S. (2007). Role of matrix metalloproteinase 13 in both endochondral and intramembranous ossification during skeletal regeneration.2 (11), e1150. 10.1371/journal.pone.0001150 PMC2063465 17987127

[B3] Berridge B. R. (2021). Animal Study Translation: the Other Reproducibility Challenge. J 62, 1–6. 10.1093/ilar/ilac005

[B4] Bhattarai G. Lee Y. H. Lee M. H. Yi H. K. (2013). Gene delivery of c-myb increases bone formation surrounding oral implants. J. Dent. Res. 92 (9), 840–845. 10.1177/0022034513497753 23838059

[B5] Bhattarai G. Lee Y. H. Lee N. H. Yun J. S. Hwang P. H. Yi H. K. (2011). c-myb mediates inflammatory reaction against oxidative stress in human breast cancer cell line, MCF-7. iochem. Funct. 29, 686–693. 10.1002/cbf.1808 21953443

[B6] Bruderer M. Richards R. G. Alini M. Stoddart M. J. (2014). Role and regulation of runx2 in osteogenesis. Eur. 23 (28), 269–286. 10.22203/ecm.v028a19 25340806

[B7] Cabral-Pacheco G. A. Garza-Veloz I. Castruita-De la Rosa C. Ramirez-Acuna J. M. Perez-Romero B. A. Guerrero-Rodriguez J. F. (2020). The roles of matrix metalloproteinases and their inhibitors in human diseases. Int. J. Mol. Sci. 21 (24), 9739. 10.3390/ijms21249739 33419373 PMC7767220

[B8] Cackowski F. C. Anderson J. L. Patrene K. D. Choksi R. J. Shapiro S. D. Windle J. J. (2010). Osteoclasts are important for bone angiogenesis. Blood 115 (1), 140–149. 10.1182/blood-2009-08-237628 19887675 PMC3988688

[B9] Checchi V. Maravic T. Bellini P. Generali L. Consolo U. Breschi L. (2020). The role of matrix metalloproteinases in periodontal disease. Int. J. Environ. Res. Public Health. 17 (14), 4923. 10.3390/ijerph17144923 32650590 PMC7399864

[B10] Cicirò Y. Sala A. (2021). MYB oncoproteins: emerging players and potential therapeutic targets in human cancer. Oncogenesis 10 (2), 19. 10.1038/s41389-021-00309-y 33637673 PMC7910556

[B11] de Wildt B. W. M. Ansari S. Sommerdijk N. A. J. M. Ito K. Akiva A. Hofmann S. (2019). From bone regeneration to three-dimensional *in vitro* models: issue engineering of organized bone extracellular matrix. Curr Opin Biomed Eng 10, 107–115. 10.1016/j.cobme.2019.05.005

[B12] Delgado-Calle J. Hancock B. Likine E. F. Sato A. Y. McAndrews K. Sanudo C. (2018). MMP14 is a novel target of PTH signaling in osteocytes that controls resorption by regulating soluble RANKL production. FASEB J 32 (5), 2878–2890. 10.1096/fj.201700919RRR 29401593 PMC5901377

[B13] Ehnert S. Rinderknecht H. Aspera-Werz R. H. Haussling V. Nussler A. K. (2020). Use of *in vitro* bone modls to screen for altered bone metabolism, osteopathies, and fracture healing: hallenges of complex models. Arch. Toxicol. 94 (12), 3937–3958. 10.1007/s00204-020-02906-z 32910238 PMC7655582

[B14] Elefteriou F. Yang X. (2011). Genetic mouse models for bone studies – strengths and limitations. Bone 49 (6), 1242–1254. 10.1016/j.bone.2011.08.021 21907838 PMC3331798

[B67] Ess K. C. Witte D. P. Bascomb C. P. Aronow B. J. (1999). Diverse developing mouse lineages exhibit high-level c-Myb expression in immature cells and loss of expression upon differentiation. Oncogene 18 (4), 1103–1111. 10.1038/sj.onc.1202387 10023687

[B15] Fatima S. Thakur S. C. (2020). New insights into the role of matrix metalloproteinases. A M B 18 (7), 1448. 10.3390/ijms18071448

[B16] Fernandez-Patron C. Kassiri Z. Leung D. (2011). Modulation of Systemic Metabolism by MMP-2: from MMP-2 Deficiency in Mice to MMP-2 Deficiency in Patients. Compr. Physiol. 6 (4), 1935–1949. 10.1002/cphy.c160010 27783864

[B17] Gaffney J. Solomonov I. Zehorai E. Sagi I. (2015). Multilevel regulation of matrix metalloproteinases in tissue homeostasis indicates their molecular specifity *in vivo* . Matrix Biol 44-46, 191–199. 10.1016/j.matbio.2015.01.012 25622911

[B18] Garcia T. Roman-roman S. Jackson A. Theilhaber J. Connolly T. Spinella-jaegle S. (2002). Behavior of osteoblast, adipocyte, and myoblast markers in genome-wide expression analysis of mouse calvaria primery osteoblasts *in vitro* . Bone 31 (1), 205–211. 10.1016/s8756-3282(02)00781-0 12110436

[B19] Haeusler G. Walter I. Helmreich M. Egerbacher M. (2005). Localization of matrix metalloproteinases, (MMPs) their tissue inhibitors, and vascular endothelial growth factor (VEGF) in growth plates of children and adolescents indicates a role for MMPs in human postnatal growth and skeletal maturation. Calcif. Tissue. Int. 76 (5), 326–335. 10.1007/s00223-004-0161-6 15868281

[B20] Howe K. M. Watson R. J. (1991). Nucleotide preferences in sequence-specific recognition of DNA by c-myb protein. Nucl. Acids. Res. 19 (14), 3913–3919. 10.1093/nar/19.14.3913 1861984 PMC328483

[B21] Hu Q. Ecker M. (2021). Overview of MMP-13 as a promising target for the treatment of osteoarthritis. Int J Mol Sci 22 (4), 1742. 10.3390/ijms22041742 33572320 PMC7916132

[B22] Inoue K. Mikuni-Takagaki Y. Oikawa K. Itoh T. Inada M. Noguchi T. (2006). A crucial role for matrix metalloproteinase 2 in osteocytic canalicular formation and bone metabolism. J. Biol. Chem. 281 (44), 33814–33824. 10.1074/jbc.M607290200 16959767

[B23] Jinnin M. (2010). Mechanisms of skin fibrosis in systemic sclerosis. J 37 (1), 11–25. 10.1111/j.1346-8138.2009.00738.x 20175837

[B24] Khoswanto Ch. (2023). Role of matrix metalloproteinases in bone regeneration: narrative review. J. Biol. Res. 13 (5), 539–543. 10.1016/j.jobcr.2023.06.002 PMC10282173 37351418

[B25] Klein T. Bischoff R. (2010). Physiology and pathophysiology of matrix metalloproteases. Acids 41 (2), 271–290. 10.1007/s00726-010-0689-x PMC3102199 20640864

[B26] Knopfova L. Benes P. Pekarcikova L. Hermanova M. Masarik M. Pernicova Z. (2012). c-Myb regulates matrix metalloproteinases 1/9, and cathepsin D: implications for matrix-dependent breast cancer cell invasion and metastasis. Mol. Cancer. 11 (1), 15–15. 10.1186/1476-4598-11-15 22439866 PMC3325857

[B27] Kodric K. Zupan J. Kranjc T. Komadina R. Mlakar V. Marc J. (2019). Sex-determining region Y (SRY) attributes to gender differences in RANKL expression and incidence of osteoporosis. Exp. Mol. Med. 51 (8), 1–16. 10.1038/s12276-019-0294-3 PMC6802671 31409771

[B28] Komori T. (2018). Runx2, an inducer of osteoblast and chondrocyte differentiation. Histochem Biol 149 (4), 313–323. 10.1007/s00418-018-1640-6 29356961

[B29] Kou L. Jiang Y. Lin X. Huang H. Wang J. Yao Q. (2021). Matrix Metalloproteinase Inspired Therapeutic Strategies for Bone Diseases. Curr. Pharm. Biotechnol. 22 (4), 451–467. 10.2174/1389201021666200630140735 32603279

[B30] Kwon M. J. (2023). Matrix metalloproteinases as therapeutic targets in breast cancer. Front Oncol 19 (12), 1108695. 10.3389/fonc.2022.1108695 PMC9897057 36741729

[B31] Laronha H. Caldeira J. (2020). Structure and function of human matrix metalloproteinases. Cells 26 (5), 1076. 10.3390/cells9051076 PMC7290392 32357580

[B32] Lee M. Partridge N. C. (2010). Parathyroid hormone activation of matrix metalloproteinase-13 transcription requires the histone acetyltransferase activity of p300 and PCAF and p300-dependent acetylation of PCAF. J Biol Chem 285 (49), 38014–38022. 10.1074/jbc.M110.142141 20870727 PMC2992235

[B33] Liang H. P. H. Xu J. Xue M. Jackson C. J. (2016). Matrix metalloproteinases in bone development and pathology: current knowledge and potential clinical utility. Med 3, 93–102. 10.2147/MNM.S92187

[B34] Liu Q. Li M. Wang S. Xiao Z. Xiong Y. Wang G. (2020). Recent advances of osterix transcription factor in osteoblast differentiation and bone formation. Front. Dev. Biol. 8, 601224. 10.3389/fcell.2020.601224 PMC7769847 33384998

[B35] Luchian I. Goriuc A. Sandu D. Covasa M. (2022). The role of matrix metalloproteinases (MMP-8, MMP-9, MMP-13) in periodontal and peri-implant pathological processes. Int J Mol Sci 23 (3), 1806. 10.3390/ijms23031806 35163727 PMC8837018

[B36] Lungova V. Buchtova M. Janeckova E. Tucker A. S. S. Smarda J. Matalova E. (2012). Localization of c-Myb in differentiated cells during postnatal molar and alveolar bone development. Eur. J. Sci. 120, 495–504. 10.1111/j.1600-0722.2012.01004.x 23167465

[B37] Matalova E. Buchtova M. Tucker A. S. Bender T. P. Janeckova E. Lungova V. (2011). Expression and characterization of c-Myb in prenatal odontogenesis. Dev. Growth Differ. 53 (6), 793–803. 10.1111/j.1440-169X.2011.01287.x 21762405

[B38] Mucenski M. L. McLain K. Kier A. B. Swerdlow S. H. Schreiner C. M. Miller T. A. (1991). A functional c-myb gene is required for normal murine fetal hepatic hematopoiesis. Cell. 65(4):677–689. 10.1016/0092-8674(91)90099-K 1709592

[B39] Nakatani T. Partridge N. C. (2017). MEF2C interacts with c-FOS in PTH-Stimulated Mmp13 gene expression in osteoblastic cells. Endocrinology 158 (11), 3778–3791. 10.1210/en.2017-00159 28973134 PMC5695834

[B40] Nishimura R. Wakabayashi M. Hata K. Matsubara T. Honma S. Wakisaka S. (2012). Osterix regulates calcification and degradation of chondrogenic matrices through matrix metalloproteinase 13 (MMP13) expression in association with transcription factor Runx2 during endochondral ossification. J. Biol. Chem. 287 (40), 33179–33190. 10.1074/jbc.M111.337063 22869368 PMC3460424

[B41] Omi M. Mishina Y. (2022). Roles of osteoclasts in alveolar bone remodeling. Genesis 60 (8-9), e23490. 10.1002/dvg.23490 35757898 PMC9786271

[B42] Oralova V. Matalova E. Janeckova E. Krejci E. D. Knopfova L. Snajdr P. (2015). Role of c-Myb in chondrogenesis. Bone 76, 97–106. 10.1016/j.bone.2015.02.031 25845979

[B43] Oralova V. Matalova E. Killinger M. Knopfova L. Smarda J. Buchtová M. (2017). Osteogenic Potential of the Transcription Factor c-MYB. Tissue Inter 100 (3), 311–322. 10.1007/s00223-016-0219-2 28012106

[B44] Owen R. Reilly G. C. (2018). *In vitro* Models of Bone Remodelling and Associated Disorders. Front Bioeng Biotechnol 11 (6), 134. 10.3389/fbioe.2018.00134 PMC6193121 30364287

[B45] Page-McCaw A. Ewald A. J. Werb Z. (2007). Matrix metalloproteinases and the regulation of tissue remodelling. Nat. Rev. Mol. Biol. 8 (3), 221–233. 10.1038/nrm2125 PMC2760082 17318226

[B46] Paiva K. B. Granjeiro J. M. (2017). Matrix metalloproteinases in bone resorption, remodeling, and repair. Prog. Mol. Biol. Transl. Sci. 148, 203–303. 10.1016/bs.pmbts.2017.05.001 28662823

[B47] Reddy S. G. Rani S. H. (2017). Matrix Metalloproteases: potential Role in Type 2 Diabetic Nephropathy. Pathophysiol, 605–616. 10.1007/978-981-10-6141-7_25

[B48] Reponen P. Sahlberg C. Munaut C. Thesleff I. Tryggvason K. (1994). High expression of 92-kD type IV collagenase (gelatinase B) in the osteoclast lineage during mouse development. J. Biol. 124 (6), 1091–1102. 10.1083/jcb.124.6.1091 PMC2119973 8132709

[B49] Robinson N. B. Krieger K. Khan F. M. Huffman W. Chang M. Naik A. (2019). The current state of animal models in research: A review. Int. J. Surg. 72, 9–13. 10.1016/j.ijsu.2019.10.015 31627013

[B50] Stickens D. Behonick D. J. Ortega N. Heyer B. Hartenstein B. Yu Y. (2004). Altered endochondral bone development in matrix metalloproteinase 13-deficient mice. Development 131 (23), 5883–5895. 10.1242/dev.01461 15539485 PMC2771178

[B51] Sandberg M. L. Sutton S. E. Pletcher M. T. Wilshire T. Tarantino L. M. Hogenesch J. B. (2005). C-Myb and p300 regulate hematopoietic stem cell proliferation and differentiation. Dev. 8 (2), 153–166. 10.1016/j.devcel.2004.12.015 15691758

[B52] Sasano Y. Zhu J. X. Tsubota M. Takahashi I. Onodera K. Mizoguchi I. (2002). Gene expression of MMP8 and MMP13 during embryonic development of bone and cartilage in the rat mandible and hind limb. J. Histochem Cytochem. 50 (3), 325–332. 10.1177/002215540205000304 11850435

[B53] Shi J. Son M. Y. Yamada S. Szabova L. Kahan S. Chrysovergis K. (2008). Membrane-type MMPs enable extracellular matrix permissiveness and mesenchymal cell proliferation during embryogenesis. Dev. Biol. 313 (1), 196–209. 10.1016/j.ydbio.2007.10.017 18022611 PMC2262846

[B54] Soza-Ried C. Hess I. Netuschil N. Boehm T. (2010). Essential role of c-myb in definitive hematopoiesis is evolutionarily conserved. PNAS 107 (40), 17304–17308. 10.1073/pnas.1004640107 20823231 PMC2951452

[B55] Svandova E. Peterkova R. Matalova E. Lesot H. (2020). Formation and developmental specification of the odontogenic and osteogenic mesenchymes. Front. Dev. Biol. 8, 640. 10.3389/fcell.2020.00640 PMC7396701 32850793

[B56] Takaishi H. Kimura T. Dala S. Okada Y. D´Armiento J. (2008). Joint diseases and matrix metalloproteinases: A role for MMP-13. Curr Pharm Biotechnol 9 (1), 47–54. 10.2174/138920108783497659 18289056

[B57] Takanche J. S. Kim J. E. Kim J. S. Lee M. H. Jeon J. G. Park I. S. (2018). Chitosan-gold nanoparticles mediated gene delivery of c-myb facilitates osseointegration of dental implants in ovariectomized rat. Artif. Cells. Biotechnol 46 (3), S807–S817. 10.1080/21691401.2018.1513940 30307328

[B58] Tang S. Y. Herber R. P. Ho S. P. Alliston T. (2012). Matrix metalloproteinase–13 is required for osteocytic perilacunar remodeling and maintains bone fracture resistance. J. Bone Min. Res. 27 (9), 1936–1950. 10.1002/jbmr.1646 PMC3415585 22549931

[B59] Vesela B. Svandova E. Bobek J. Lesot H. Matalová E. (2019). Osteogenic and angiogenic profiles of mandibular bone-forming cells. Front. Phys. 10, 124. 10.3389/fphys.2019.00124 PMC6389724 30837894

[B60] Vincent-Chong V. K. Salahshourifar I. Karen-Ng L. P. Siow Y. M. Kallarakkal T. G. Ramanathan A. (2014). Overexpression of MMP13 is associated with clinical outcomes and poor prognosis in oral squamous cell carcinoma. Sci World J 2014, 897523. 10.1155/2014/897523 PMC4226172 25401159

[B61] Visse R. Nagase H. (2003). Matrix metalloproteinases and tissue inhibitors of metalloproteinases: structure, function, and biochemistry. 92 (8), 827–839. 10.1161/01.RES.0000070112.80711.3D 12730128

[B62] Vu J. L. Werb Z. (2000). Matrix metalloproteinases: effectors of development and normal physiology. Dev 14 (17), 2123–2133. 10.1101/gad.815400 10970876

[B63] Ward E. (2022). A Review of Tissue Engineering for Periodontal Tissue Regeneration. J Vet Dent 39 (1), 49–62. 10.1177/08987564211065137 34935526

[B64] Xu L. H. Zhao F. Yang W. W. Chen C. W. Du Z. H. Fu M. (2019). Myb promotes the growth and metastasis of salivary adenoid cystic carcinoma. Int J Oncol 54 (5), 1579–1590. 10.3892/ijo.2019.4754 30896785 PMC6438425

[B65] Young D. A. Barter M. J. Wilkinson D. J. (2019). Recent advances in understanding the regulation of metalloproteinases. eCollection 18 (8), F1000 Faculty Rev-195. 10.12688/f1000research.17471.1 PMC6381797 30828429

[B66] Zhang C. Tang W. Li Y. (2012). Matrix metalloproteinase 13 (MMP13) is a direct target of osteoblast-specific transcription factor osterix (Osx) in osteoblasts.7 (11), e50525. 10.1371/journal.pone.0050525 PMC3503972 23185634

