# Supplementary figures and images for: Expression dynamics of metalloproteinases during mandibular bone formation: association with Myb transcription factor
Source: Front Cell Dev Biol. 2023 Aug 28;11:1168866. doi: 10.3389/fcell.2023.1168866 (PMC10493412; doi:10.3389/fcell.2023.1168866)

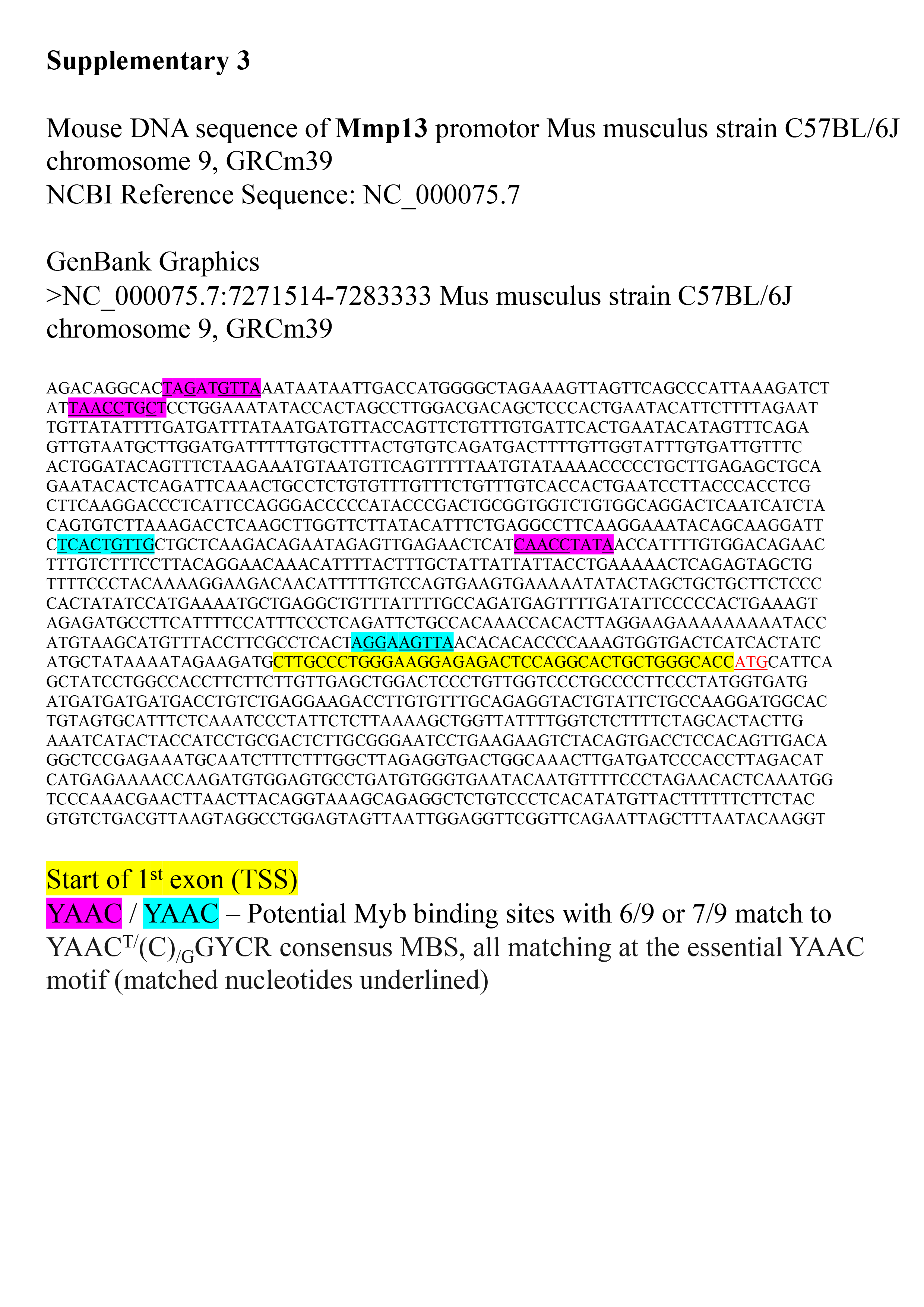

Supplement: Supplementary file 1 [file Image3.TIF]

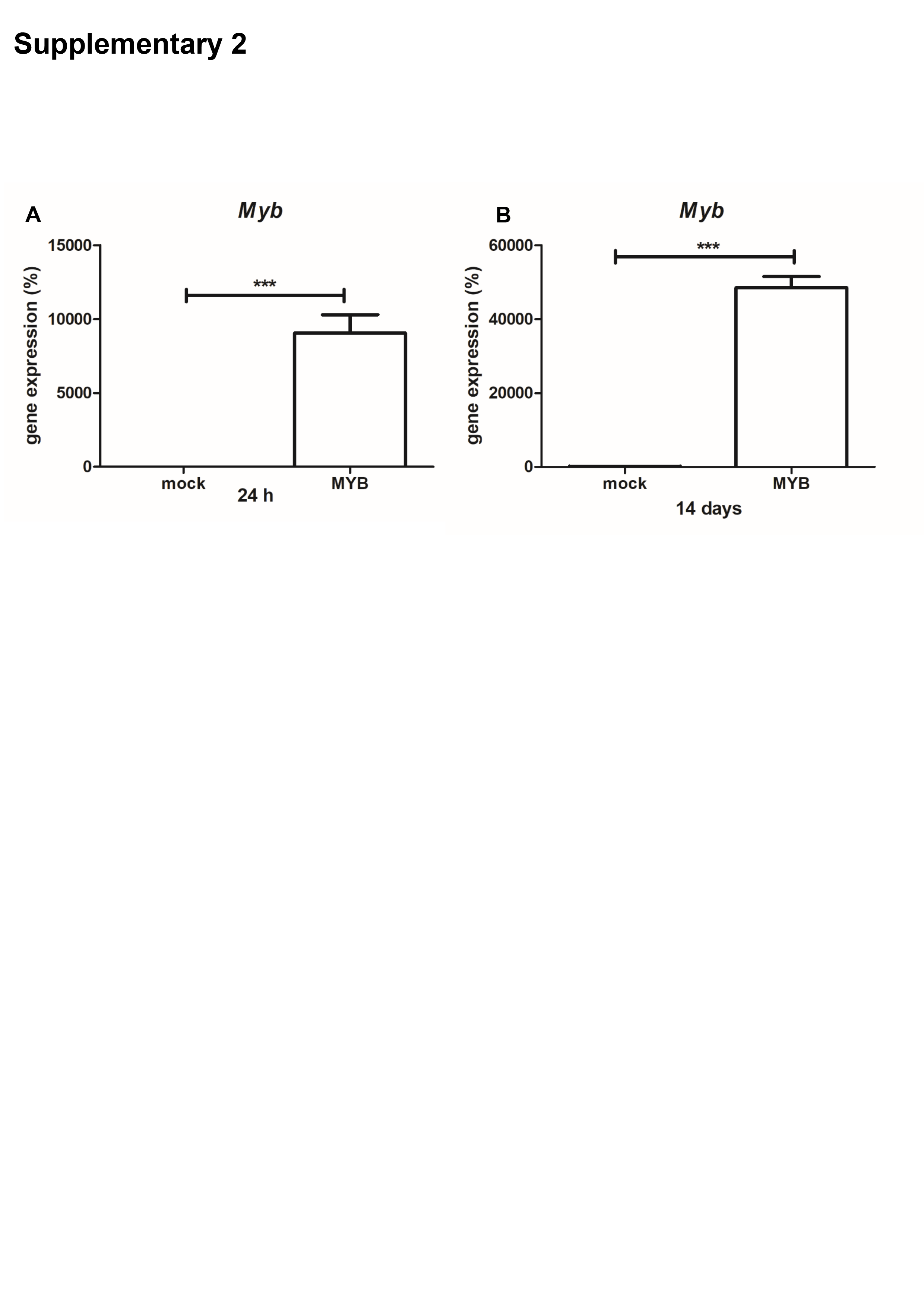

Supplement: Supplementary file 2 [file Image2.TIF]

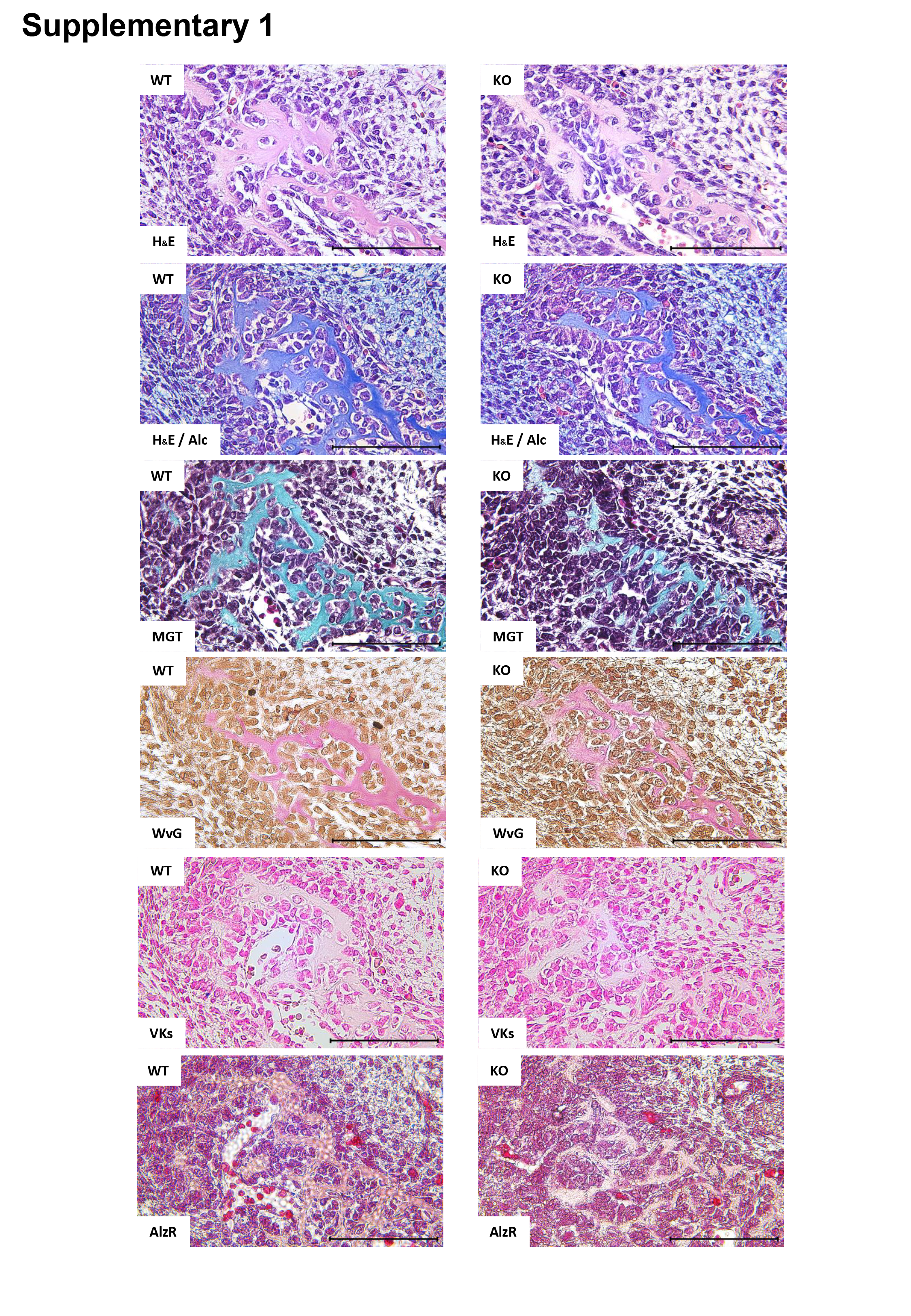

Supplement: Supplementary file 3 [file Image1.TIF]
